# Comparing Metabolomic and Essential Oil Fingerprints of *Citrus australasica* F. Muell (Finger Lime) Varieties and Their In Vitro Antioxidant Activity

**DOI:** 10.3390/antiox11102047

**Published:** 2022-10-18

**Authors:** Emily Cioni, Chiara Migone, Roberta Ascrizzi, Beatrice Muscatello, Marinella De Leo, Anna Maria Piras, Ylenia Zambito, Guido Flamini, Luisa Pistelli

**Affiliations:** 1 Dipartimento di Farmacia, Università di Pisa, via Bonanno 33, 56126 Pisa, Italy; 2 Centro Interdipartimentale di Ricerca “Nutraceutica e Alimentazione per la Salute”, Via del Borghetto 80, Università di Pisa, 56124 Pisa, Italy; 3 Centro per l’Integrazione della Strumentazione dell’Università di Pisa (CISUP), Lungarno Pacinotti 43, 56126 Pisa, Italy

**Keywords:** *Citrus australasica*, finger lime, phenols, volatiles, antioxidant, UHPLC-MS/Orbitrap, chemometrics

## Abstract

Comparative chemical analyses among peel and pulp essential oils (EOs) and methanolic extracts of four *Citrus australasica* varieties (Red, Collette, Pink Ice, and Yellow Sunshine), and the hybrid *Faustrime*, were performed using GC-MS and UHPLC-DAD-HR-Orbitrap/ESI-MS. Peel and pulp extracts were also analysed for their in vitro antioxidant activity on a Balb/3T3 clone A31 mouse embryo fibroblast cell line. The results of peel and pulp EOs were mainly characterised by monoterpenes and sesquiterpenes, respectively. All peels displayed a higher total phenol content (TPC) than pulps, and consequently a greater antioxidant activity. Collette peels and Pink Ice pulps showed the highest amount of identified flavonoids (e.g., luteolin, isosakuranetin, and poncirin derivatives). Collette and Red peels were rich in anthocyanins (delphinidin and petunidin glycosides), exhibiting the maximum protective activity against induced oxidative damage. In conclusion, finger lime fruits are good sources of health-promoting phytocomplexes, with the Red, Collette, and Pink Ice varieties being the most promising.

## 1. Introduction

The *Citrus* genus, belonging to the Rutaceae family, includes widely distributed, consumed, and studied species, such as *Citrus limon* (L.) Osbeck (lemon), *C. medica* L. (cedar), *C. × aurantium* L. (bitter orange), *C. paradisi* Macfad. (grapefruit), *C. reticulata* Blanco (tangerine), and *C. sinensis* (L.) Osbeck (orange), but also the lesser known *C. australasica* F. Muell [1,2]. *Citrus australasica* is a small tree native to Australia which is recently acquiring growing commercial interest in Italy and in Europe, in general, due to the uniqueness of its fruits that are used in gourmet culinary preparations. *Citrus australasica* is commonly called finger lime or lemon caviar, because its spindle-shaped fruits are finger-like, while the vesicles of its pulp are similar to pearls of caviar. There are several varieties and hybrids of *C. australasica* that differ macroscopically in peel and pulp color, and have more or less acidulous or floral odorous notes [3]. These differences reflect changes in qualitative and quantitative chemical composition, and may mean a greater or lesser content of bioactive compounds and/or secondary metabolites of therapeutic interest. Previous evidence reports that *Citrus* fruits are a source of both macronutrients (e.g., simple sugars, fibers, and water) and micronutrients (e.g., folic acid, thiamine, niacin, vitamin C, and vitamin B6). Their pulps, peels, and seeds contain minerals (potassium, calcium, phosphorus, and magnesium), and are free of sodium and cholesterol; moreover, they are low in proteins and fats [4]. However, their secondary metabolites are the constituents of major interest, especially flavonoids (flavones, flavonols, flavanones, and flavanonols), phenolic acids (hydroxybenzoic acids and hydroxycinnamic acids), anthocyanins, coumarins, and limonoids, which have demonstrated antioxidant, anti-inflammatory, anti-cancer, and neuro-cardioprotective activities [5,6]. Among the flavonoids, the most common aglycones in the *Citrus* genus are naringenin, hesperetin, apigenin, nobiletin, tangeretin, and quercetin, often carrying saccharide chains that are composed of glucose, rhamnose, rutinose, and neohesperidose. Sinapic, *p*-coumaric, ferulic, caffeic, and gallic acids are the most common phenolic acids that have been identified in the fruits of this genus. Furthermore, among the bitter limonoid constituents, limonin, nomilin, obacunone, and limonexic acid are also present [7].

More than 170 molecules with antioxidant activity have been identified in the most common fruits of the *Citrus* genus. The antioxidant activity could be attributed to the whole phytocomplex, which is capable of ensuring the maintenance of healthy cell structure and function through the inactivation of free radicals, the inhibition of lipid peroxidation, and the prevention of harmful oxidative mechanisms [8]. Free radicals, or reactive oxygen species (ROS), by-products of the metabolism of aerobic cells, can generate other peroxidic and hydroperoxidic radicals that are capable of interacting with lipid molecules, or, in a cytotoxic manner, with nucleic acids and proteins essential for life, damaging them or altering their functionality. Therefore, the search for molecules that are capable of counteracting these free radicals is of great interest [7,9].

In this context, *C. australasica* fruits attract great attention as a potential source of bioactive molecules with antioxidant properties. To the best of our knowledge, few current studies have been reported in the literature, with most focused on volatile components of Alstonville, Judy’s Everbearing, and Durham’s Emerald varieties, along with *Faustrime* hybrid [10,11]; total phenolic content of four Florida-grown selections [3]; the phenolic composition of XiangBin and LiSiKe varieties [2]; and a Spanish cultivated plant [12].

**Figure 1:**
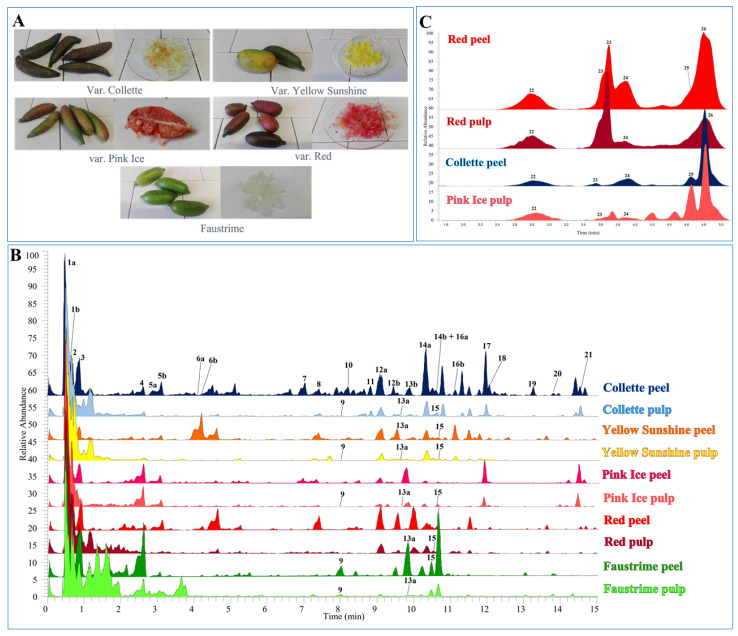
(**A**) *Citrus australasica* studied varieties; (**B**) comparison of chromatograms of *C. australasica* peels and pulps extracts, recorded by UHPLC-HR-Orbitrap/ESI-MS analyses in negative ion mode; (**C**) anthocyanin HR-Orbitrap/ESI-MS profiles of *C. australasica* peels and pulps, recorded in positive ion mode.

Based on the previous promising studies and the growing economic, gastronomic, and health-related demands on this peculiar *Citrus* fruit, the aim of the present study was to carry out a full comparative chemical analysis on both volatile and non-volatile components of the peel and pulp of *C. australasica* varieties, with particular attention to phenolic compounds and anthocyanins, still lacking in the literature. To this purpose, four *C. australasica* varieties (Collette, Yellow Sunshine, Pink Ice, and Red), and the hybrid species *Faustrime* (*Monocitrus australasica* × *Fortunella* sp. × *Citrus aurantifolia*) (Figure 1A), were selected. The metabolomic profile of all varieties was investigated by means of ultra-high performance liquid chromatography (UHPLC), coupled with a diode array detector (DAD) and a high-resolution Orbitrap-based electrospray ionization source mass spectrometer (HR-Orbitrap/ESI-MS); meanwhile, the EO composition was established through gas chromatography coupled with mass spectrometry (GC/MS). All of the extracts were also investigated for their in vitro antioxidant activity on a Balb/3T3 clone A31 mouse embryo fibroblast cell line. This study is part of a larger project conducted by our research group, which is aimed at re-evaluating the beneficial properties of fruits of the genus *Citrus*, due to their high content of bioactive compounds belonging to the class of polyphenols and triterpenoids [13,14,15,16,17].

## 2. Materials and Methods

### 2.1. Chemicals and Reagents

UHPLC-grade *n*-hexane, acetonitrile, methanol, water, and formic acid Supelco^®^ were purchased from Merck KGaA (Darmstadt, Germany). All analytical-grade solvents were purchased from VWR (Milano, Italy). Standards of rutin, hesperidin, and gallic acid were purchased from Merck KGaA (Darmstadt, Germany), cyanidin 3-*O*-glucoside chloride was purchased from Extrasynthese (Extrasynthese, France), and luteolin-4′-*O*-neohesperidoside was previously obtained in our laboratory by isolation from plant materials and characterised by 1D- and 2D-NMR techniques. Folin–Ciocâlteu reagent was purchased from Merck KGaA (Darmstadt, Germany). The Balb/3T3 clone A31 mouse embryo fibroblast cell line was purchased from the American Type Culture Collection LGC standards (ATCC CCL163, Milan, Italy) and propagated as indicated by the supplier. Dulbecco’s Modified Eagle’s medium (DMEM), 0.01 M pH 7.4 phosphate buffer saline without Ca^2+^ and Mg^2+^ (PBS), bovine calf serum (BCS), glutamine, and antibiotics (penicillin/streptomycin) were obtained from Merck KGaA (Darmstadt, Germany). Cell proliferation reagent WST-1 was provided by Roche Diagnostic (Milan, Italy).

### 2.2. Plant Materials and Non-Volatile Extract Preparation

The fruits of *C. australasica* varieties (Red, Collette, Pink Ice, and Yellow Sunshine), and the hybrid species *Faustrime* (Figure 1A), were provided by the Agrumi Lenzi Company (Pescia, Pistoia, Italy) in October 2019. Fruits (6 for each variety) were collected at the ripening stage from plants that were growing in pots. For each fruit, peels were separated from pulps. For the extraction of non-volatile compounds, peels were dried in an oven at 40 °C, while pulps were freeze-dried (Modulyo, Pirani 501, Edwards, UK). The dried material was stored at room temperature, and protected from light until extraction. A portion of fresh material was stored at −20 °C for essential oil preparation (see Section 2.5) and anthocyanin extraction.

For each variety, powdered peels and pulps were first defatted with *n*-hexane, then subjected to extraction with methanol (solid:liquid mg/mL ratio 1:20 *w*/*v*) via dynamic maceration (120 rpm) with a digital orbital shaker (IKA™ KS 501, Minerva S.r.l., Pisa, Italy) for three consecutive days at room temperature, renewing the solvent every 24 h. Finally, the solvent was removed under vacuum to obtain dry extracts, as reported in Table S1.

Anthocyanins were extracted from the peels of Red and Collette varieties, and from the pulps of Red and Pink Ice varieties, and characterised by visible pigmentation. For extraction, 500 mg of defrosted plant material were placed in 3 mL of a 2% methanol/hydrochloric acid mixture for 15 min under stirring, then centrifuged for 5 min at 4000 rpm. The supernatants were withdrawn using a syringe, and directly analysed in triplicate using UHPLC-HR-ESI-MS.

### 2.3. Determination of the Total Polyphenol Content

Total polyphenol content (TPC) was evaluated in the methanolic extracts of *C. australasica* peels and pulps following the colorimetric method of Folin–Ciocâlteu [18]. Methanol solutions (10 mg/mL) of peel and pulp extracts were diluted with water until a final concentration of 0.48 mg/mL was reached for peels and 0.70 mg/mL for pulps. Samples were prepared by adding 1 mL of distilled water, 100 μL of Folin–Ciocâlteu reagent, and 300 μL of Na_2_CO_3_ (20%) to 500 μL of the aqueous solutions; then, the samples were mixed and incubated in the dark at room temperature for 2 h. The absorbance was measured at 765 nm against a blank solution using a UV-VIS spectrophotometer (Lambda 25, Perkin-Elmer, Waltham, MA, USA). Gallic acid was employed as the standard in the concentration range 0.005–0.030 mg/mL (*R*^2^ = 0.999). All of the samples were tested in quadruplicate, and the results were expressed as mg of gallic acid equivalents (GAE)/g of dry weight (DW) [19].

### 2.4. UHPLC-DAD-HR-Orbitrap/ESI-MS Analyses

#### 2.4.1. Quali-Quantitative Analyses of Phenols

Quali-quantitative chemical analyses were performed via UHPLC using a Vanquish Flex Binary pump that was coupled with a DAD and an HR Q Exactive Plus MS, based on Orbitrap technology, equipped with an ESI source, a hybrid-quadrupole analyser and Xcalibur 3.1 software (Thermo Fischer Scientific Inc., Bremem, Germany). Elutions were conducted at a flow rate of 0.5 mL/min, using a splitting system of 1:1 to an MS detector (250 μL/min) and a DAD/UV detector (250 μL/min), respectively.

For each variety of *C. australasica*, methanol extracts were solubilized in methanol at concentrations of 2 and 10 mg/mL for the peels and the pulps, respectively. These solutions were prepared in triplicate, and centrifuged for 5 min at 4000 rpm in order to remove suspended particles. Volumes of 5 μL of the supernatants were injected into the LC-MS system. Chromatographic analyses were performed using a 2.1 × 100 mm, 2.6 µm, Kinetex^®^ Biphenyl C-18 column provided from a Security Guard^TM^ Ultra Cartridge (Phenomenex, Bologna, Italy), and a mixture of HCOOH in H_2_O 0.1% *v*/*v* (solvent A) and acetonitrile in H_2_O 0.1% *v*/*v* (solvent B) as the mobile phase. A linear gradient was used, increasing from 5 to 35% B in 15 min for both the methanol extracts of peels and pulps. DAD data were recorded in a 200–600 nm range, with the three preferential channels set at 254, 280, and 325 nm, which are typical absorbances for phenolic compounds. The HR-MS results were acquired in a *m*/*z* scan range of 250–1200 in negative ion mode, operating in full (resolution 70,000 and maximum injection time of 220 ms) and data dependent-MS/MS (resolution 17,500 and maximum injection time of 60 ms). The used ionization parameters were the following: nebulization voltage of 3500 V, capillary temperature of 300 °C, sheath gas (N_2_) 20 arbitrary units, auxiliary gas (N_2_) 3 arbitrary units, and an HCD (higher-energy C- trap dissociation) of 18 eV.

In order to quantify the phenolic compounds that were identified in the five methanol extracts of *C. australasica* varieties, four calibration curves were constructed using rutin as the external standard for flavonol glycosides, hesperidin for flavanones glycosides, luteolin 4′-*O*-neohesperidoside for flavone glycosides, and chlorogenic acid for hydroxycinnamic acids and their esters. Stock solutions of 1 mg/mL of each standard were prepared, and then different concentrations were obtained using serial dilutions. Rutin, hesperidin, and luteolin 4′-*O*-neoesperidoside were prepared in triplicate acetonitrile solutions in a concentration range of 15.63–1.95 μg/mL, while concentrations of 200–20 μg/mL were used for the chlorogenic acid standard. Integration of the peak areas obtained for each standard in UHPLC-HR-MS was related to the respective concentration, and the equation of the resulting curve was used to quantify the phenolic compounds. The obtained curves showed a good linearity in the range of prepared concentrations and correlation coefficients (*R*^2^) equal to 0.993 for rutin, 0.990 for hesperidin, 0.995 for luteolin 4′-*O*-neoesperidoside, and 0.999 for chlorogenic acid, were obtained. The amount of each compound was calculated using Microsoft^®^ Office Excel, and expressed as μg/g of dried peel or freeze-dried pulp (DW) ± standard deviation.

#### 2.4.2. Quali-Quantitative Analyses of Anthocyanins

The anthocyanin extracts were analysed in triplicate using UHPLC-DAD-HR-ESI-MS. The solutions (5 μL injection volume) were injected in a 2.1 × 100 mm, 2.6 µm, Kinetex^®^ Biphenyl C-18 column provided by a Security Guard^TM^ Ultra Cartridge (Phenomenex, Bologna, Italy), at a flow rate of 0.5 mL/min. A mixture of HCOOH in H_2_O 0.1% *v*/*v* (solvent A) and acetonitrile in H_2_O 0.1% *v*/*v* (solvent B) was used for the elution, according to a linear gradient from 5 to 20% B in 5 min. UV data were recorded using 515 nm as a detection wavelength, the typical absorbance of anthocyanins. The HR-MS data were acquired in a *m*/*z* scan range of 120–1200 in positive ion mode, operating in full and data dependent-MS/MS using the same ionization parameters as for phenols.

In order to quantify the anthocyanins in the peels of the Collette and Red varieties, as well as in the pulps of Red and Pink Ice varieties, a calibration curve was constructed with cyanidin 3-*O*-glucoside as an external standard. Triplicate acetonitrile solutions at the concentrations of 0.05, 0.025, and 0.0025 μg/mL were prepared, beginning from a stock 1 mg/mL solution. By correlating the integrations of the peak areas with the respective standard concentrations, a curve was obtained that showed good linearity in the selected concentration range, and an *R*^2^ equal to 0.996. The amount of the anthocyanins identified in the plant material was obtained by Microsoft^®^ Office Excel, and finally expressed as μg/g of dried peel or freeze-dried pulp (DW) ± standard deviation.

### 2.5. Essential Oils (EOs) Hydrodistillation and Analysis

For all of the samples, 15 g of defrosted peels and 30 g of fresh pulps (after removal of the seeds) were subjected to hydrodistillation in a standard Clevenger apparatus for 2 h. The hydrodistillation duration was experimentally determined as the time necessary for the complete EO volatilisation from the samples. For each sample, triplicates were performed. The hydrodistillation yields could not be evaluated, given the small material amount; thus, the volatile fraction was captured in HPLC-grade *n*-hexane in the Clevenger apparatus. The EOs in HPLC-grade *n*-hexane were stored in amber-glass vials and maintained at –20 °C until analysis.

The hydrodistilled samples were injected into a GC-MS apparatus. Gas chromatography–electron impact mass spectrometry (GC-EIMS) analyses were performed with an Agilent 7890B gas chromatograph (Agilent Technologies Inc., Santa Clara, CA, USA) that was equipped with an Agilent HP-5MS (Agilent Technologies Inc., Santa Clara, CA, USA) capillary column (30 m × 0.25 mm; coating thickness 0.25 μm) and an Agilent 5977B single quadrupole mass detector (Agilent Technologies Inc., Santa Clara, CA, USA). The analytical conditions used were as follows: injector and transfer line temperatures 220 and 240 °C, respectively; oven temperature programmed from 60 to 240 °C at 3 °C/min; carrier gas helium at 1 mL/min; injection of 1 μL (0.5% HPLC grade *n*-hexane solution); split ratio 1:25. The acquisition parameters used were as follows: full scan; scan range: 30–300 *m*/*z*; scan time: 1.0 sec. The identification of the constituents was based on a comparison of their retention times with those of authentic samples (when available), comparing their linear retention indices relative to the series of *n*-hydrocarbons. Computer matching was also used against a commercial [20] and a laboratory-developed mass spectra library that was built up from pure substances and components of commercial essential oils of known composition, and from MS literature data [21].

### 2.6. Cell Viability

Cell viability evaluations of *C. australasica* extracts were performed using the Balb/3T3 clone A31 cell line. Cells were grown in complete DMEM containing 10% bovine calf serum (BCS), 4 mM glutamine, and 100 U/mL:100 μg/mL penicillin:streptomycin. Balb/3T3 clone A31 fibroblast cells were seeded in 96-well culture plates at a concentration of 10^4^ cells per well, incubated at 37 °C and 5% CO_2_, and left to proliferate for 24 h prior to the incubation with the samples. The culture medium from each well was removed and replaced with a medium containing pre-dissolved sample in dimethyl sulfoxide (DMSO), and diluted with complete DMEM at different concentrations. Cells incubated with fresh growth medium were used as a control. The DMSO percentage in control and extract samples was kept at 1% *v*/*v*. With a view to the assessment of antioxidant effects, cytotoxicity ranges of peel and pulp extracts were set on GAE equivalents, resulting in 15–120 μg/mL for peels, and 30–300 μg/mL for pulps. After 2 h of incubation, cell viability was assessed using WST-1 tetrazolium salt reagent diluted to 1:10, and incubated for 4 h at 37 °C and 5% CO_2_. Measurements of formazan dye absorbance were carried out at 450 nm, with the reference wavelength of 655 nm, using a microplate reader (BioTek 800/TS, Thermo Scientific).

### 2.7. Cell Treatment and Oxidative Stress

Adherent Balb/3T3 fibroblast cells, grown on 96-well culture plates, were incubated for 2 h with peel and pulp extracts that were diluted to polyphenol concentrations of 0.25, 0.50, and 1.00 μg/mL GAE in complete DMEM (Table S2). After the treatment, the cells were washed with phosphate buffered saline (PBS), and stressed with 1500 μM of commercial H_2_O_2_ for 1 h. Fibroblast cells incubated with H_2_O_2_ without sample treatment were considered as reference for the oxidative stress. The cells were evaluated for viability by means of WST-1 reagent. Cell viability percentages were referred to Balb/3T3 control cells, in the absence of treatment and without H_2_O_2_ incubation [22].

### 2.8. Statistical Analyses

All of the analyses were performed with JMP^®^ Pro 14.0.0 (SAS Institute Inc., Cary, NC, USA) software.

For the statistical evaluation of all the EO compositions, an 89 × 10 correlation matrix (89 individual compounds × 10 samples = 890 data) was used, while for composition of phenols and anthocyanins, a 28 × 10 correlation matrix (28 individual compounds × 10 samples = 280 data) was applied. In order to perform the principal component analysis (PCA), linear regressions were operated on mean-centred, unscaled data to select the two highest principal components (PCs). This unsupervised method reduced the dimensionality of the multivariate data of the matrix, whilst preserving most of the variance [23]. For the essential oil analyses, the chosen PC1 and PC2 explained 58.5% and 20.0% of the variance, respectively, for a total explained variance of 78.5%. For the non-volatile components, the chosen PC1 and PC2 explained 52.9% and 25.1% of the studied variance, respectively, for a total studied variance of 78.0%. A hierarchical cluster analysis (HCA) was performed using Ward’s method, with Euclidean distances as a measure of similarity. The observations of the groups of samples performed with HCA and the PCA unsupervised methods can be applied even when there are no available reference samples that can be used as a training set to establish the model.

The significant difference (*p* value < 0.05) between groups of values was evaluated using a one-way ANOVA.

## 3. Results and Discussion

### 3.1. Polyphenol Content of Finger Lime Fruits

The TPC was determined for both peels and pulps of the four varieties of *C. australasica* (Red, Collette, Pink Ice, and Yellow Sunshine), and the hybrid species *Faustrime* (Table 1). In general, the results highlighted significantly different TPC among the varieties, but always a higher content of polyphenols in peels than pulps. Specifically, Red and Pink Ice peels had similar TPC values that were higher than the other varieties (9.1 ± 0.2 and 8.2 ± 0.2 mg of GAE/g of DW, respectively). Meanwhile, the hybrid species *Faustrime* was the most lacking in TPC, as shown by the quantitative datum 4.9 ± 0.1 mg of GAE/g DW. Regarding the pulps, Pink Ice and Collette were the two varieties that were most abundant in polyphenols (6.4 ± 0.2 and 5.6 ± 0.2 mg of GAE/g DW, respectively); *Faustrime* was confirmed to be poor in TPC, as was Yellow Sunshine variety (3.1 ± 0.2 and 2.6 ± 0.1 mg of GAE/g DW, respectively). Even though Yellow Sunshine peels had a TPC value that was comparable to the other *C. australasica* varieties, a significant decrease was observed in the pulps (Table 1). Compared to previous results reported by [12], all of the extracts showed higher TPC levels in both peels and pulps.

### 3.2. Metabolomic Fingerprint and Quantitative Analysis of Non-Volatile Components

#### 3.2.1. Phenol Composition

The quali-quantitative analyses of *C. australasica* fruits were performed using UHPLC-DAD-HR-Orbitrap/ESI-MS technique. The chromatographic profiles of all peels and pulps showed similarities and differences, both within the same variety and among different varieties (Figure 1B).

The tentative identification of constituents was performed by comparing their elution order, UV data, HR full mass spectra, and fragmentation patterns, with data that were reported in the literature [2,24]. The level of the identification led to the proposal of tentative candidates, since it was not possible to establish the position of substituents based only on full MS and MS/MS experiments. In addition, a mass error < 5 ppm on the experimental molecular formula was considered for the annotation. Following this approach, 7 hydroxycinnamic acid derivatives, 18 glycosylated flavonoids, and a limonoid, were tentatively identified from all of the analysed finger lime fruits (Table 2). Compounds **7**, **16**, and **24** were confirmed on the basis of injection of reference standards.

In the first chromatographic region (0–5 min; Figure 1B), hydroxycinnamic acid derivatives were characterised. Compounds **1a** and **1b** (*t*_R _ = 0.50 and 0.66 min) are two caffeoylisocitric acid isomers, as indicated by HR mass data, showing the deprotonated ion [M-H]^−^ at *m*/*z* 353.0723, the ion product [M-162-H]^−^ at *m*/*z* 191.02 corresponding to isocitric acid that was generated by the loss of 162 u due to the cleavage of an ester bond with a caffeic acid residue. Compound **2** was annotated as caffeoylmethylisocitric acid, due to the deprotonated ion [M-H]^−^ at *m*/*z* 367.0879, and the presence of a methylisocitric product ion at *m*/*z* 205.03 that was generated by the loss of a caffeoyl residue (−162 u). These hydroxycinnamic acid tricarboxylic acid esters are metabolites that are not commonly found in plants, especially isocitric acid derivatives [25]. Compounds **5a** and **5b** were identified as two *p*-coumaroylglucoside acid isomers. In the mass spectrum recorded in full scan, a parent ion at *m*/*z* 325.0927 was observed for both molecules, which under collision energy lost a hexose residue, and generated a *p*-coumaroyl fragment at *m*/*z* 163.04. Compounds **6a** and **6b** showed the same deprotonated ion [M-H]^−^ at *m*/*z* 355.1034 in the full MS, while in the MS/MS experiment a feruloyl ion product at *m*/*z* 193.05 that was generated by the cleavage of the glycosidic bond was observed; thus, the two compounds were tentatively attributed to feruloylglucoside acid isomers. Compounds **3** and **4** were not fully identified, but information about a portion of the molecule was deduced by the analyses of their MS fragmentation patterns. Compound **3** ([M-H]^−^ at *m*/*z* 433.0596) showed product ions that were in common with compound **2** at *m*/*z* 205.03, 143.03, and 111.00, indicating the occurrence of a methylisocitric acid derivative. Compound **4** ([M-H]^−^ at *m*/*z* 365.1451) showed the presence of typical product ions at *m*/*z* 303.14, 263.11, and 221.10, due to the loss of 62, 102, and 144 u, respectively, indicating the occurrence of a 3-hydroxy-3-methylglutaric acid derivative.

In the chromatographic region within 7–15 min, nine flavonol glycosides (compounds **7**, **8**, **11**, **12a**, **12b**, **14a**, **14b**, **18**, and **19**), seven flavanone glycosides (compounds **9**, **13a**, **13b**, **16a**, **16b**, **17**, and **21**), and two flavone glycosides (compounds **10** and **15**) were found. Both compounds **7** and **8** displayed the flavonol quercetin (*m*/*z* 301.04) as an aglycon portion. In particular, compound **7** was identified as rutin, as deduced by the parent ion at *m*/*z* 609.1458 and the observed loss of a rutinose residue (308 u); meanwhile, for compound **8** (deprotonated ion [M-H]^−^ at *m*/*z* 463.0880), a loss of a hexose unit was observed, suggesting the occurrence of a quercetin glucoside. Compounds **11** and **19** were kaempferol derivatives, as indicated by the product ion at *m*/*z* 285.04 in the MS/MS. Compound **11** (deprotonated molecular ion [M-H]^−^ at *m*/*z* 447.1035) was annotated as a kaempferol glucoside, due to the loss of a hexose residue (−162 u), while for compound **19** ([M-H]^−^ at *m*/*z* 771.2354), a kaempferol triglucoside structure was suggested ([M-162-162-162-H] ^−^ at *m*/*z* 285.04). Compounds **12a**, **12b**, **14a**, **14b**, and **18** all exhibited a base ion peak at *m*/*z* 315.04 in the MS/MS, which was attributed to isorhamnetin. Compounds **12a** and **12b** ([M-H]^−^ at *m*/*z* 477.1035) were assigned as isorhamnetin glucoside isomers, showing the loss of a hexose unit. Compounds **14a** and **14b** showed the same deprotonated ion at *m*/*z* 621.1457, and diagnostic fragments for a hexose unit (−162), and a 3-hydroxy-3-methylglutaryl residue (−62, −102, −144 u). Compound **18** ([M-H]^−^ at *m*/*z* 765.1881) differed from **14a** and **14b,** only for having one more unit of 3-hydroxy-3-methylglutaric acid; thus, it was annotated as a di-(3-hydroxy-3-methylglutaryl) isorhamnetin glucoside. Compound **9** was revealed only in the fruits of the hybrid species *Faustrime*, and it could correspond to neoeriocitrin or eriocitrin, two flavanones glycosides that are commonly found in the genus *Citrus* [9]. In addition to the deprotonated ion [M-H]^−^ at *m*/*z* 595.1666, a base ion peak at *m*/*z* 287.06 that corresponded to the aglycone portion of eriodictyol was observed, due to the loss of a disaccharide (308 u) which could be attributed to a rutinose or a neohesperidose, since they cannot be distinguished only on the basis of mass spectra. Peaks **13a** and **13b** displayed the same parent ion at *m*/*z* 579.1713 and the same base ion peak at *m*/*z* 271.02 attributed to naringenin. The loss of a disaccharide unit [M-H–308]^−^ due to a rutinose or neohesperidose residue suggested the presence of two isomers, tentatively identified as naringin (naringenin neohesperidoside) and naringenin rutinoside. Compounds **16a** and **16b** were two isomeric forms of the same molecule, as deduced from the same deprotonated molecular ion [M-H]^−^ at *m*/*z* 609.1029, and from the overlapping fragmentation mass spectra in which the base ion peak at *m*/*z* 301.07 was attributed to hesperetin. The two isomers were annotated as neohesperidin (hesperetin neohesperidoside) and hesperidin (hesperetin rutinoside). Peak **17** ([M-H]^−^ at *m*/*z* 755.2415) was tentatively identified as isosakuranetin rhamnosyldiglucoside, since in the ESI-MS/MS, a base ion peak at *m*/*z* 285.08 ([M-162-162-146-H] ^−^) generated by the loss of two hexose residues (probably glucose) and a deoxyhexose (probably rhamnose) was observed. Poncirin (**21**, [M-H]^−^ at *m*/*z* 593.1878) displayed a fragment ion at *m*/*z* 285.08 that was assigned to isosakuranetin, previously reported in *C. australasica* by Wang et al. (2019). The full MS ([M-H]^−^ at *m*/*z* 593.1514) and MS/MS (base ion peak at *m*/*z* 285.04) suggested compound **10** as luteolin 7-*O*-neohesperidoside or luteolin 7-*O*-rutinoside, according to the aforementioned previous study (Wang et al., 2019). Compound **15** exhibited a parent ion at *m*/*z* 607.1666, and a diagnostic product ion at *m*/*z* 299.06, which was assigned to diosmetin. For its glycosidic portion, the option between two disaccharides (308 u), neohesperidose and rutinose, was considered; therefore, **15** could be annotated as diosmin or neodiosmin. Compound **20** ([M-H]^−^ at *m*/*z* 501.1763) was identified as limonexic acid, which belongs to the class of limonoids, typical terpenoids of the genus *Citrus* and responsible for their bitter taste [26]. The fragmentation peaks observed in the ESI-MS/MS at *m*/*z* 457.18 and 413.20 are in agreement with the data reported in the literature [27].

From a qualitative point of view, our results confirmed the presence of some components that were identified in *C. australasica* peel and pulp via UHPLC-MS/MS from a previous study [12]. To the best of our knowledge, the chemical composition of the hybrid species *Faustrime* has herein been reported for the first time. There are differences among the five varieties, especially in the case of the hybrid species *Faustrime* (Table 2), which showed several typical constituents of the *Citrus* genus, such as (neo)eriocitrin, (neo)diosmin, and naringenin rutinoside/naringin, that are rarely found in the other *C. australasica* varieties.

The quantitative estimation of all of the constituents (Table 3) that were obtained through UHPLC-MS highlighted greater differences among all studied fruits. Generally, it was confirmed that all phenol constituents are more abundant in peels than in pulps. The hydroxycinnamic acid derivatives were present in peels and the pulps of all varieties in very similar amounts, with the exception of Pink Ice, which was particularly rich in caffeoylisocitric acid. The largest amount of total flavonoids found among peels was in Collette (3432 ± 239 μg/g dry weight, DW), and in Pink Ice among pulps (897 ± 43 μg/g DW). Considering the whole fruit, among the identified compounds, the most abundant ones were rutin, luteolin 7-*O*-neohesperidoside/rutinoside, isosakuranetin rhamnosyldiglucoside, and poncirin, in Collette and Pink Ice; quercetin glucoside, isorhamnetin glucoside, and neohesperidin in Yellow Sunshine; quercetin glucoside, naringin, and poncirin in Red. The hybrid species *Faustrime* was distinguished by the significant presence, especially in the peels, of (neo)eriocitrin (316 ± 27 μg/g DW), (neo)diosmin (606 ± 41 μg/g DW), and naringenin rutinoside/naringin (145 ± 12 μg/g DW).

#### 3.2.2. Anthocyanins Characterisation

Anthocyanins were identified in the extracts that were obtained from Red and Collette peels, and from Red and Pink Ice pulps, by comparing the data obtained through UHPLC-UV-ESI-MS/MS (Figure 1C) with those from a previous study on the *Citrus* fruits [28]. Five anthocyanins derived from cyanidin, delphinidin, petunidin, and peonidin were found (compounds **22–26**, Table 2).

Compound **22** (*t*_R_ = 2.78 min) was characterised as cyanidin 3-*O*-glucoside, as deduced by ESI-MS/MS data showing a molecular ion [M]^+^ at *m*/*z* 449.1068, and a base ion peak at *m*/*z* 287.05 that corresponded to the aglycone portion of cyanidin, and generated by the loss of a hexose residue (−162 u). Compound **23** (*t*_R_ = 3.68 min) was assigned a molecular weight equal to 787.2272 u, on the basis of molecular ion [M]^+^ recorded in full scan MS. In the fragmentation spectrum, a base ion peak at *m*/*z* 317.06 ([M-162-146-162]^+^) was observed, corresponding to the aglycone portion, petunidin, generated by the loss of two hexose and one deoxyhexose residues; thus, **23** was tentatively identified as petunidin rhamnosyldiglucoside. Cyanidin 3-(6″-malonylglucoside) (**24**, *t*_R_ = 3.87 min) was characterised by a molecular ion [M]^+^ at *m*/*z* 535.1071, and a base ion peak at *m*/*z* 287.05 that was attributed to the aglycone, cyanidin, generated by the loss of a malonyl residue and a hexose [M-162-86]^+^. Peonidin 3-(6″-malonylglucoside) (**25**, *t*_R_ = 4.85 min) displayed a molecular ion [M]^+^ at *m*/*z* 549.1227, a product ion at *m*/*z* 301 corresponding to peonidin, and similarly to compound **24**, the loss of 86 and 162 u residues. Compound **26** (*t*_R_ = 4.76 min, [M]^+^ = 611.1593) was annotated as delphinidin rhamnosylglucoside. The analysis of the fragmentation pattern highlighted the aglycone portion at *m*/*z* 303.05, which was identified as delphinidin, and the loss of a disaccharide ([M-162-146]^+^) was attributable to hexose and deoxyhexose residues.

Based on the quantitative analysis (Table 3), cyanidin 3-*O*-glucoside was found to be the most representative anthocyanin, both in peels and pulps, of all of the investigated varieties. In agreement with our results, cyanidin 3-*O*-glucoside was found to be the most abundant anthocyanin from a previous study of red finger lime [29]. The peels of the Collette variety were the richest in anthocyanins (143.2 ± 3.2 μg/g DW), followed by the peels (96.2 ± 2.3 μg/g DW) and the pulps (8.26 ± 0.56 μg/g DW) of the Red variety.

#### 3.2.3. Multivariate Statistical Analyses of the Non-Volatile Components

**Figure 2:**
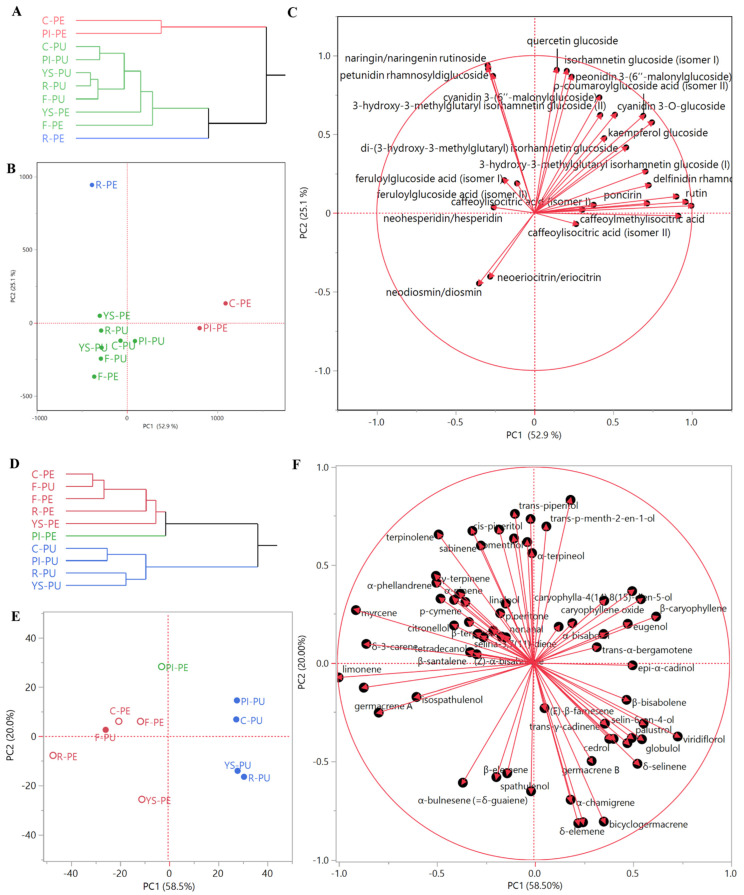
Non-volatile (**A**) and EO (**D**) dendrograms of the hierarchical cluster analysis (HCA), and respective score (**B**,**E**) and loadings (**C**,**F**) plots of the principal component analysis (PCA) performed on peels (PE) and pulps (PU) for essential oil and non-volatile compositions of all of the samples (C = Collette; F = Faustrime; PI = Pink Ice; R = Red; YS = Yellow Sunshine).

The dendrogram of the HCA (Figure 2A) shows a sample distribution into two macro-clusters: the first one (red samples) comprised Collette and Pink Ice peels, while the second one comprised two clusters (blue and green). The blue cluster is composed only of Red peel, while the green cluster includes the pulps of all of the varieties, as well as the *Faustrime* and Yellow Sunshine peels.

This organ-driven statistical distribution was confirmed through the PCA. Collette and Pink Ice peels were plotted on the right quadrants (PC1 > 0) of the score plot (Figure 2B). Red peels were plotted in the right area of the upper left quadrant (PC1 < 0, PC2 > 0) (score plot, Figure 2B), due to their high anthocyanin content (loadings plot, Figure 2C). The pulps of all of the varieties and of Yellow Sunshine peels were plotted on the left quadrants (PC1 < 0) of the score plot (Figure 2B), due to their content of neodiosmin/diosmin and neoeriocitrin/eriocitrin. Pink Ice and Yellow Sunshine varieties were plotted along the PC1 axis, and in the upper region of the bottom quadrants (PC2 < 0) (score plot, Figure 2B), due to their content of caffeoylisocitric acid (isomers I and II) and hydroxycinnamic acid derivatives (loadings plot, Figure 2C).

### 3.3. Essential Oil (EO) Composition of All of the Samples

The complete composition of all of the EOs that were hydrodistilled from both the peels and pulps of all the *C. australasica* varieties (Collette, Pink Ice, Red, and Yellow Sunshine), and the *Faustrime* hybrid, are reported in Table 4. Overall, 89 compounds were identified from the EO compositions.

Monoterpenes were the most abundant compounds found in all of the peel EOs, with the exception of the Yellow Sunshine variety, where sesquiterpenes prevailed. The Collette and Red varieties, as well as the *Faustrime* peel EOs, can be considered a limonene chemotype, whereas the Pink Ice and the Yellow Sunshine EOs exhibited a 4-terpineol/limonene and a limonene/bicyclogermacrene chemotype, respectively. This difference in peel EO chemotypes was found to be consistent with previous literature studies for other finger lime varieties [10,30,31], although the latter analysed a dichloromethane extract of the volatile peel constituents. Among the monoterpenes, their hydrocarbon form prevailed in all of the peel EOs, with the exception of the Pink Ice variety, where the oxygenated monoterpenes were more abundant. Among the monoterpene hydrocarbons, limonene was found to be the most quantitatively relevant in all of the samples, accounting for up to 73.6% in the Red variety. With the exception of the Red and Yellow Sunshine varieties, γ-terpinene and α-phellandrene followed, as relative concentrations. Among the peel EOs, oxygenated monoterpenes were more abundant in the Pink Ice variety and the *Faustrime* hybrid, exhibiting a relative presence of 48.0 and 35.5%, respectively. Among this class, 4-terpineol was observed to be the most abundant in the former, while citronellal and piperitone prevailed in the latter. This quantitatively relevant presence of citronellal and piperitone in the *Faustrime* hybrid EO is in accordance with [32], although their analysis was performed with an essential oil that was obtained by peel cold-pressing, and [11]. Bicyclogermacrene was the sesquiterpene hydrocarbon that exhibited the highest relative abundance in all of the peel EOs. Sesquiterpenes dominated all of the pulp EO compositions, with the only exception being the *Faustrime* hybrid, whose composition was mainly represented by monoterpenes. The Yellow Sunshine pulp EO was mainly composed of oxygenated sesquiterpenes, among which viridiflorol and globulol were the most represented; however, like the Red variety, the most characterising compound in its composition was bicyclogermacrene, a sesquiterpene hydrocarbon. Among the latter chemical classes, β-caryophyllene was detected as the main relevant compound in the Collette variety, while it exhibited a comparable presence to its oxidized counterpart (caryophyllene oxide) in the leaf EO of the Pink Ice variety. α-Humulene and β-bisabolene followed within this class, reaching up to 12.7% in the Pink Ice pulp EO and 24.6% in the Red variety pulp EO, respectively. Viridiflorol, globulol, and guaiol were detected as the most abundant oxygenated sesquiterpenes in the pulp EOs of all of the analysed samples, with the exception of the *Faustrime* hybrid. The latter was, indeed, chiefly composed of monoterpene hydrocarbons, which represented over 70% of its complete composition, with limonene being the most abundant compound (48.3%), followed by γ-terpinene (10.8%), and α-phellandrene (7.2%).

#### Multivariate Statistical Analyses of the EO Compositions

The dendrogram of the HCA (Figure 2D) evidenced a distribution of the samples into two macro-clusters: the first comprised two clusters (red and green) and included all of the peel Eos, and the *Faustrime* pulp sample; the second macro-cluster was homogeneous (blue samples), and comprised all of the *C. australasica* pulp EOs, except the *Faustrime* pulp.

This organ-driven statistical distribution was confirmed with principal component analysis. All of the peel EOs were plotted on the left quadrants (PC1 < 0) of the score plot (Figure 2E), together with the *Faustrime* pulp sample. The Pink Ice peel EO was plotted in the right area of the upper left quadrant (PC1 < 0, PC2 > 0) (score plot, Figure 2E), due to its 4-terpineol content (loadings plot, Figure 2F). Collette and *Faustrime* peel EOs were closely grouped towards the center of the same quadrant, very close to the *Faustrime* pulp EO (score plot, Figure 2E): all of these samples were grouped in this area due to the contribution of γ-terpinene and α-phellandrene (loadings plot, Figure 2F). Both the Red and Yellow Sunshine peel EOs were plotted in the bottom right quadrant (PC1 and PC2 > 0) of the PCA score plot (Figure 2E): as evidenced in the loadings plot (Figure 2F), their position was due to their high relative content of limonene. With the exception of the *Faustrime* hybrid sample, all of the pulp EOs were plotted in the right quadrants (PC1 > 0) of the PCA score plot (Figure 2E). Collette and Pink Ice pulp EOs were grouped in the upper quadrant (PC2 > 0): the positioning of the former was mainly due to its high relative content of caryophyllene oxide, while β-caryophyllene and α-humulene showed a high relative presence in both samples. Finally, Yellow Sunshine and Red pulp EOs were plotted in the bottom right quadrant (PC1 > 0, PC2 < 0) of the PCA score plot (Figure 2E), due to the contributions of bicyclogermacrene, globulol, viridiflorol, and guaiol vectors (loadings plot, Figure 2F).

### 3.4. Cell Viability Assay and In Vitro Cellular Assessment of Antioxidant Properties

**Figure 3:**
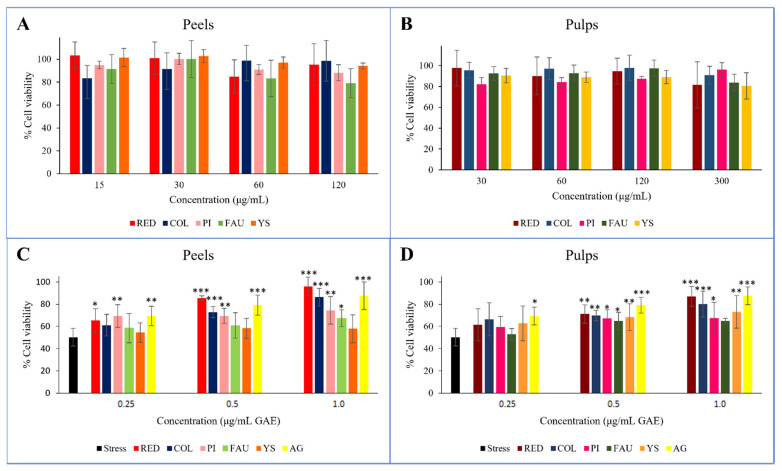
In vitro cell evaluation of the Balb/3T3 cell line. Cytotoxicity screening after 2-h treatments with peel (**A**) and pulp (**B**) extracts. Protective effects of peel (**C**) and pulp (**D**) extracts from H_2_O_2_-induced oxidative stress, reported as cell viability % after 2-h treatments with 1500 μM H_2_O_2_ of pre-treated Balb/3T3 cells. H_2_O_2_ = stressed control; C = Collette; F = *Faustrime*; GA = gallic acid; PI = Pink Ice; R = Red; YS = Yellow Sunshine; *** = *p* value < 0.0001; ** = *p* value < 0.005; * = *p* value < 0.05 vs. stress.

Before evaluating the extent of the protection they provided from oxidative stress, the cytotoxicity on Balb/3T3 of the peel and pulp extracts was assayed. The concentration ranges were set on the basis of relevant GAE equivalents, resulting in 15–120 μg/mL for peels, and 30–300 μg/mL for pulps. Neither the peel nor pulp extracts showed cytotoxic effects at any of the tested concentrations (Figure 3A,B).

The antioxidant protective effect was assessed in vitro for all peel (Figure 3C) and pulp (Figure 3D) extracts. Three different concentrations were evaluated, namely 0.25, 0.50, and 1.0 μg/mL GAE, and compared to gallic acid that was used as a reference. The oxidative treatment with H_2_O_2_ resulted in a drastic decrease in cell viability (45%) with respect to untreated and unstressed control cells. Cell viability was increased with the pretreatment of all extracts, thus protecting cells from the induced oxidative stress. In general, the observed effects were directly proportional to the corresponding concentration of gallic acid, with greater accordance recorded for the Red and Collette extracts. In these cases, the effects on cell viability correlated with increasing GAE concentration treatments, incrementing from 65 to 96%, and from 61 to 87% for Red peel and pulp, respectively; and from 61 to 86%, and from 66 to 80% for Collette peel and pulp, respectively. Concerning the hybrid species *Faustrime*, a poor protection effect was detected for both its peel and pulp. This evidence could be explained on the basis of the higher polyphenol content in the Red and Collette varieties, and moreover, due to the valuable amounts of anthocyanins in these varieties.

## 4. Conclusions

The chemical investigation of the four finger lime varieties revealed an interesting profile of potentially health-promoting agents that were represented by hydroxycinnamic acids (ferulic, *p*-coumaric, and caffeic acid derivatives) and glycosylated flavonols (kaempferol, quercetin, and isorhamnetin derivatives), flavanones (naringenin, eriodictyol, and hesperetin derivatives), and flavones (luteolin and diosmetin derivatives). Furthermore, the glycosides of cyanidin, delfinidin, petunidin, and peonidin were the anthocyanins that were detected in the Red, Pink Ice, and Collette varieties. Among limonoids, triterpenoids typically found in *Citrus* fruits that are responsible for their bitter taste, only limonexic acid was revealed. For each variety, the peel and pulp showed similar qualitative profiles; among all of the samples, the hybrid species *Faustrime* differed for the presence of neoeriocitrin, eriocitrin, neodiosmin, and diosmin, components that are usually predominant in other common *Citrus* fruits. All of the peels differed from their relative pulps in terms of phenol amount; they were richer in bioactive components, as confirmed by their higher antioxidant capacity observed in their provided protection from oxidative damage. Similarly, the volatile compositions of peel EOs of all of the samples were characterised mainly by monoterpenes, while pulp EOs were rich in sesquiterpenes.

The uniqueness of the organoleptic characteristics of these fruits, jointly with their composition that is rich in antioxidant metabolites, make them promising candidates for their use as fresh fruits, or for the development of nutraceutical products with beneficial properties.

## Figures and Tables

**Table 1:** Total polyphenol content (TPC) of *Citrus australasica* peel and pulp extracts, expressed as mg of gallic acid equivalents (mg GAE) for mass of dry weight (g DW) ± standard deviation (SD).

Finger Lime Varieties	Peel	Pulp
	mg GAE/g DW ± SD	mg GAE/g DW ± SD
Red	9.1 ± 0.2	5.0 ± 0.2
Collette	7.4 ± 0.2	5.6 ± 0.2
Pink Ice	8.2 ± 0.2	6.4 ± 0.2
Yellow Sunshine	6.8 ± 0.2	2.6 ± 0.1
Faustrime	4.9 ± 0.1	3.1 ± 0.2

**Table 2:** Chromatographic data (retention time, *t*_R_) and HR-ESI-MS/MS data of compounds **1–26**, detected in peels and pulps of *Citrus australasica* F. Muell. C = Collette; F = *Faustrime*; PI = Pink Ice; R = Red; YS = Yellow Sunshine. pe = only peel; pu = only pulp.

N. ^a^	Compound	*t_R_*(min)	[M-H]^−^	Formula	Error (ppm)	-ESI-MS/MS (*m*/*z*) ^b^	Extract
	*Hydroxycinnamic acids*						
**1a**	Caffeoylisocitric acid (isomer I)	0.50	353.0723	C_15_H_14_O_10_	−0.8497	293.05; 191.02; 173.01; **111.01**	R; F; PI; C; YS
**1b**	Caffeoylisocitric acid (isomer II)	0.66	353.0723	C_15_H_14_O_10_	−0.8497	173.01; 120.20; **111.01**; 87.01	R; F; PI; C; YS
**2**	Caffeoylmethylisocitric acid	0.7–2	367.0879	C_16_H_16_O_10_	−0.8172	205.03, 191.02; 179.06; 169.01; 161.05; 143.03; **111.00**; 101.02; 89.02	R; F; PI; C; YS
**3**	Methylisocitric acid derivative	0.88	433.0596	-	-	401.04; 227.02; 205.03; 173.01; 143.03; **111.01**; 87.01	R; F; PI; C; YS
**4**	3-Hydroxy-3-methylglutaric acid derivative	2.62	365.1451	-	-	303.14; 263.11; 221.10; 161.04; 125.02; 99.04; 59.87; **57.03**	R; F; PI; C
**5a**	*p*-Coumaroylglucoside acid (isomer I)	2.87	325.0927	C_15_H_18_O_8_	−0.9228	163.04; 159.05; **145.03**	R; F (pe); PI; C; YS
**5b**	*p*-Coumaroylglucoside acid (isomer II)	3.11	325.0927	C_15_H_18_O_8_	−0.9228	163.04; 159.05; **145.03**	R; F(pe); PI; C; YS
**6a**	Feruloylglucoside acid (isomer I)	3.99	355.1034	C_16_H_20_O_9_	−0.2816	193.05; **175.04**; 160.02	R; F(pe); PI; C; YS
**6b**	Feruloylglucoside acid (isomer II)	4.19	355.1034	C_16_H_20_O_9_	−0.2816	193.05; **175.04**; 160.02	R; F(pe); PI; C; YS
	*Flavonoids*						
**7**	Rutin	7.02	609.1458	C_27_H_30_O_16_	−0.4925	301.04; 300.03; 271.03	R; F; PI; C; YS
**8**	Quercetin glucoside	7.33	463.0880	C_21_H_20_O_12_	−0.4319	301.04; **300.03**	R; F; PI; C; YS
**9**	Neoeriocitrin/eriocitrin	7.99	595.1666	C_27_H_32_O_15_	−0.3360	459.11; **287.06**; 193.01; 161.02; 151.00; 135.04	F; PI(pu); C(pu); YS(pu)
**10**	Luteolin 7-*O*-neohesperidoside/rutinoside	8.19	593.1514	C_27_H_30_O_15_	+0.3372	529.27; 474.31; **285.04**; 182.91	R; F (pu); PI; C; YS
**11**	Kaempferol glucoside	8.47	447.0933	C_21_H_20_O_11_	0	327.05; 304.33; 285.04; **284.03**; 256.04; 255.03; 227.03	R; F(pe); PI; C; YS
**12a**	Isorhamnetin glucoside (isomer I)	9.07	477.1035	C_22_H_22_O_12_	−0.8384	357.06; 327.06; 315.05; 314.04; 286.05; 285.04; 271.02; 257.05; 243.03	R; F; PI; C; YS
**12b**	Isorhamnetin glucoside (isomer II)	9.52	477.1035	C_22_H_22_O_12_	−0.8384	449.11; 357.06; 333.73; 315.04; 299.02; 285.04; 271.02; 243.03	R; F; PI; C; YS
**13a**	Naringin/Naringenin rutinoside	9.50	579.1713	C_27_H_32_O_14_	−1.0360	471.43; 397.56; 313.07; 295.06; 285.08; **271.06**; 151.00	F; PI(pu); C(pu); YS
**13b**	Naringin/Naringenin rutinoside	9.94	579.1713	C_27_H_32_O_14_	−1.0360	313.07; **271.06**; 151.00	R; F; PI; C; YS
**14a**	3-Hydroxy-3-methylglutaryl isorhamnetin glucoside (isomer I)	10.30	621.1457	C_28_H_30_O_16_	−0.6440	596.51; 559.15; 519.11; 477.10; **315.05**; 299.02; 285.04; 271.02; 243.03	R; F; PI; C; YS
**14b**	3-Hydroxy-3-methylglutaryl isorhamnetin glucoside (isomer II)	10.59	621.1458	C_28_H_30_O_16_	−0.6440	559.15; 519.11; 477.10; **315.05**; 300.03; 271.03	R; F; C; PI; YS
**15**	Neodiosmin/diosmin	10.46	607.1666	C_28_H_32_O_15_	−0.3294	341.07; 299.06; 284.03; 266.07; 255.03; 151.00	R(pu); F; PI(pu); C(pu); YS
**16a**	Neohesperidin/hesperidin	10.65	609.1820	C_28_H_34_O_15_	−0.8208	418.95; 343.08; **301.07**; 286.05; 151.00	R; F; PI; C; YS
**16b**	Neohesperidin/hesperidin	11.01	609.1820	C_28_H_34_O_15_	−0.8208	**301.07**; 286.05; 151.00	R; F; PI; C(pu); YS
**17**	Isosakuranetin rhamnosildiglucoside	11.93	755.2415	C_34_H_44_O_19_	+1.4565	771.95; 755.24; 657.34; 490.63; **285.08**	R; F; PI; C; YS
**18**	Di-(3-hydroxy-3-methylglutaryl) isorhamnetin glucoside	12.03	765.1881	C_34_H_38_O_20_	−0.3921	678.23; 642.82; 621.15; 519.12; 477.11; **315.05**; 299.02; 271.03; 187.04; 151.00	R; F; PI; C; YS
**19**	Kaempferol triglucoside	13.29	771.2354	C_34_H_44_O_20_	+0.1297	527.23; 499.11; 408.51; **285.08**; 251.90	PI; C
**21**	Poncirin	14.47	593.1878	C_28_H_34_O_14_	+0.1564	593.19; 427.38; 327.09; **285.08**	R; F; PI; C; YS
	*Limonoids*						
**20**	Limonexic acid	13.76	501.1763	C_26_H_30_O_10_	−0.5986	**457.18**; 413.20; 271.89; 145.08	F; C; YS
*Anthocyanins*							
**N.^a^**	**Compound**	***t_R_*** **(min)**	**[M]^+^**	**Formula**	**Error** **(ppm)**	**+ESI-MS/MS** **(*m*** **/*z*** **)**	**Extract**
**22**	Cyanidin 3-*O*-glucoside	2.78	449.1068	C_21_H_21_O_11_^+^	−2.2266	**287.05**; 241.05; 213.05; 185.06; 157.06	R(pe, pu); C; PI
**23**	Petunidin rhamnosyldiglucoside	3.68	787.2272	C_34_H_43_O_21_^+^	−2.4135	625.17; 479.12; 427.10; **317.06**; 302.04	R(pe, pu)
**24**	Cyanidin 3-(6′′-malonylglucoside)	3.87	535.1071	C_24_H_23_O_14_^+^	−2.0557	**287.05**; 241.05; 213.05; 171.04	R(pe, pu); C
**25**	Delfinidin rhamnosylglucoside	4.76	611.1593	C_27_H_31_O_16_^+^	−2.1271	366.30; **303.05**; 203.84; 173.85	R(pe); C; PI
**26**	Peonidin 3-(6′′-malonylglucoside)	4.85	549.1227	C_25_H_25_O_14_^+^	−2.0032	517.09; 449.11; 301.07; **287.05**; 241.05; 213.05	R(pe, pu); C; PI

^a^ Compounds are listed in ascending order of retention time; the numbering of the compounds corresponds to that used in Figure 1. All compounds were tentatively identified based on MS data, except for rutin (**7**), hesperidin (**16**), and cyanidin 3-*O*-glucoside (**24**), which were confirmed via injection of reference standards. ^b^ The base ion peak is indicated in bold.

**Table 3:** Content of phenolic compounds (μg/g of dried peel or pulp ± standard deviation) and anthocyanins (μg/g of fresh peel or pulp ± standard deviation) in *C. australasica* peel and pulp extracts. nd = not determined.

		Variety					
Peak ^a^	Compound		Collette	Yellow Sunshine	Pink Ice	Red	*Faustrime*
**1a**	Caffeoylisocitric acid (isomer I)	Peel	1.18 ± 0.19 ^C^	1.45 ± 0.05 ^B^	2.01 ± 0.0 ^A^	1.23 ± 0.0 ^BC^	0.837 ± 0.026 ^D^
		Pulp	0.911 ± 0.039 ^D^	1.47 ± 0.06 ^B^	2.23 ± 0.03 ^A^	1.03 ± 0.03 ^C^	0.919 ± 0.011 ^D^
**1b**	Caffeoylisocitric acid (isomer II)	Peel	0.226 ± 0.031 ^C^	0.283 ± 0.013 ^B^	0.354 ± 0.012 ^A^	0.212 ± 0.004 ^C^	0.156 ± 0.014 ^D^
		Pulp	0.215 ± 0.022 ^C^	0.379 ± 0.029 ^B^	0.451 ± 0.026 ^A^	0.179 ± 0.008 ^CD^	0.159 ± 0.004 ^D^
**2**	Caffeoylmethylisocitric acid	Peel	0.179 ± 0.023 ^A^	0.143 ± 0.002 ^B^	0.167 ± 0.002 ^A^	0.153 ± 0.006 ^B^	0.172 ± 0.003 ^A^
		Pulp	0.114 ± 0.006 ^D^	0.143 ± 0.002 ^C^	0.329 ± 0.000 ^A^	0.071 ± 0.02 ^C^	0.069 ± 0.001 ^B^
**5a**	*p*-Coumaroylglucoside acid (isomer I)	Peel	0.105 ± 0.008 ^A^	0.043 ± 0.007 ^C^	0.092 ± 0.009 ^AB^	0.080 ± 0.05 ^B^	0.0066 ± 0.0003 ^D^
		Pulp	0.0072 ± 0.0005 ^BC^	0.0092 ± 0.001 ^B^	0.059 ± 0.006 ^A^	0.0083 ± 0.0004 ^B^	Not detected ^C^
**5b**	*p*-Coumaroylglucoside acid (isomer II)	Peel	0.187 ± 0.008 ^A^	0.089 ± 0.005 ^B^	0.189 ± 0.022 ^A^	0.171 ± 0.004 ^A^	0.012 ± 0.001 ^D^
		Pulp	0.013 ± 0.001 ^B^	0.016 ± 0.001 ^B^	0.118 ± 0.006 ^A^	0.018 ± 0.000 ^B^	Not detected ^C^
**6a**	Feruloylglucoside acid (isomer I)	Peel	0.011 ± 0.000 ^C^	0.198 ± 0.018 ^A^	0.013 ± 0.001 ^C^	0.036 ± 0.002 ^B^	0.0033 ± 0.0001 ^C^
		Pulp	0.0015 ±0.0001 ^D^	0.0031 ± 0.003 ^C^	0.010 ± 0.000 ^A^	0.0061 ± 0.0002 ^B^	Not detected ^E^
**6b**	Feruloylglucoside acid (isomer II)	Peel	0.064 ± 0.008 ^B^	0.388 ± 0.032 ^A^	0.021 ± 0.003 ^B^	0.060 ± 0.003 ^B^	0.017 ± 0.002 ^B^
		Pulp	0.0030 ±0.0002 ^CD^	0.0045 ± 0.0005 ^BC^	0.025 ± 0.003 ^A^	0.0068 ± 0.0001 ^B^	Not detected ^D^
**7**	Rutin	Peel	198 ± 13 ^A^	18.7 ± 2.0 ^C^	128 ± 12 ^B^	5.69 ± 0.49 ^C^	19.6 ± 1.3 ^C^
		Pulp	7.50 ± 0.29 ^B^	2.64 ± 0.21 ^D^	21.1 ± 1.7 ^A^	0.534 ± 0.136 ^D^	4.72 ± 0.65 ^C^
**8**	Quercetin glucoside	Peel	105 ± 5 ^C^	145 ± 12 ^B^	106 ± 10 ^C^	245 ± 9 ^A^	2.32 ± 0.03 ^D^
		Pulp	28.6 ± 1.4 ^A^	11.7 ± 0.8 ^B^	32.0 ± 7.5 ^A^	12.3 ± 0.2 ^B^	0.775 ± 0.050 ^C^
**9**	Neoeriocitrin/eriocitrin	Peel	Not detected ^B^	Not detected ^B^	Not detected ^B^	Not detected ^B^	316 ± 27 ^A^
		Pulp	0.488 ± 0.109 ^B^	1.38 ± 0.06 ^B^	1.06 ± 0.05 ^B^	Trace ^B^	56.8 ± 2.8 ^A^
**10**	Luteolin 7-*O*-neohesperidoside/rutinoside	Peel	660 ± 50 ^A^	14.7 ± 1.2 ^C^	224 ± 19 ^B^	7.72 ± 0.35 ^C^	Not detected ^C^
		Pulp	19.1 ± 0.9 ^B^	4.87 ± 0.25 ^C^	35.7 ± 2.7 ^A^	1.55 ± 0.06 ^C^	38.0 ± 2.4 ^A^
**11**	Kaempferol glucoside	Peel	11.8 ± 0.8 ^C^	14.3 ± 1.4 ^C^	76.0 ± 7.2 ^A^	43.8 ± 2.2 ^B^	2.55 ± 0.13 ^D^
		Pulp	1.99 ± 0.12 ^BC^	1.06 ± 0.06 ^C^	10.7 ± 0.8 ^A^	2.89 ± 0.05 ^B^	Not detected ^D^
**12a**	Isorhamnetin glucoside (isomer I)	Peel	288 ± 14 ^B^	171 ± 11 ^C^	47.8 ± 3.9 ^D^	376 ± 21 ^A^	5.15 ± 0.31 ^E^
		Pulp	69.3 ± 2.3 ^A^	32.2 ± 1.0 ^C^	44.1 ± 2.6 ^B^	65.0 ± 1.2 ^A^	3.31 ± 0.09 ^D^
**12b**	Isorhamnetin glucoside (isomer II)	Peel	37.6 ± 1.2 ^C^	161 ± 10 ^B^	15.5 ± 1.0 ^D^	234 ± 4 ^A^	1.04 ± 0.06 ^E^
		Pulp	15.4 ± 0.9 ^B^	20.9 ± 0.8 ^A^	7.86 ± 0.57 ^C^	20.5 ± 0.4 ^A^	0.426 ± 0.031 ^D^
**13a**	Naringin/naringenin rutinoside	Peel	Not detected ^B^	9.65 ± 0.72 ^B^	Not detected ^B^	Trace ^B^	145 ± 12 ^A^
		Pulp	0.176 ± 0.049 ^C^	4.60 ± 0.20 ^B^	0.379 ± 0.050 ^C^	Not detected ^C^	13.6 ± 0.5 ^A^
**13b**	Naringin/naringenin rutinoside	Peel	21.9 ± 1.9 ^C^	146 ± 12 ^B^	22.0 ± 2.4 ^C^	1061 ± 63 ^A^	2.26 ± 0.25 ^C^
		Pulp	15.1 ± 0.5 ^BC^	17.4 ± 1.1 ^B^	13.5 ± 0.8 ^C^	131 ± 1 ^A^	Trace ^D^
**14a**	3-Hydroxy-3-methylglutaryl isorhamnetin glucoside (I)	Peel	573 ± 24 ^A^	92.3 ± 5.8 ^B^	36.1 ± 2.5 ^C^	102 ± 2 ^B^	7.53 ± 0.37 ^C^
		Pulp	108 ± 6 ^A^	31.4 ± 1.6 ^C^	48.7 ± 3.2 ^B^	22.9 ± 0.3 ^C^	5.79 ± 0.25 ^D^
**14b**	3-Hydroxy-3-methylglutaryl isorhamnetin glucoside (II)	Peel	67.0 ± 4.2 ^A^	30.2 ± 1.8 ^C^	4.02 ± 0.34 ^D^	42.9 ± 2.2 ^B^	1.39 ± 0.10 ^D^
		Pulp	25.8 ± 1.5 ^A^	9.53 ± 0.55 ^B^	5.21 ± 0.46 ^C^	10.3 ± 0.3 ^B^	0.553 ± 0.008 ^D^
**15**	Neodiosmin/diosmin	Peel	Not detected ^C^	79.7 ± 7.5 ^B^	Not detected ^C^	Trace ^C^	606 ± 41 ^A^
		Pulp	1.70 ± 0.18 ^C^	49.6 ± 3.1 ^B^	4.41 ± 1.10 ^C^	1.39 ± 0.07 ^C^	198 ± 8 ^A^
**16a**	Neohesperidin/hesperidin	Peel	2.11 ± 0.20 ^B^	74.6 ± 7.4 ^B^	2.61 ± 0.13 ^B^	4.21 ± 1.03 ^B^	1495 ± 107 ^A^
		Pulp	4.72 ± 1.20 ^C^	48.5 ± 1.7 ^B^	6.52 ± 0.49 ^C^	3.31 ± 0.14 ^C^	174 ± 5 ^A^
**16b**	Neohesperidin/hesperidin	Peel	Trace ^B^	502 ± 43 ^A^	2.92 ± 0.10 ^B^	18.0 ± 1.9 ^B^	10.5 ± 2.6 ^B^
		Pulp	1.53 ± 0.43 ^B^	107 ± 7 ^A^	1.67 ± 0.11 ^B^	4.43 ± 0.18 ^B^	0.471 ± 0.149 ^B^
**17**	Isosakuranetin rhamnosyldiglucoside	Peel	1119 ± 102 ^A^	13.4 ± 1.2 ^C^	911 ± 92 ^B^	2.21 ± 0.00 ^C^	4.09 ± 0.45 ^C^
		Pulp	157 ± 4 ^B^	2.17 ± 0.12 ^C^	272 ± 13 ^A^	1.49 ± 0.09 ^C^	0.348 ± 0.029 ^C^
**18**	Di-(3-hydroxy-3-methylglutaryl) isorhamnetin glucoside	Peel	114 ± 10 ^A^	28.2 ± 2.2 ^C^	4.11 ± 0.12 ^D^	41.2 ± 1.1 ^B^	11.6 ± 0.4 ^D^
		Pulp	10.1 ± 0.6 ^A^	9.61 ± 0.58 ^A^	6.57 ± 0.53 ^B^	6.20 ± 0.01 ^B^	3.87 ± 0.22 ^C^
**19**	Kaempferol triglucoside	Peel	11.8 ± 0.8 ^A^	Trace ^C^	9.85 ± 0.82 ^B^	Not detected ^C^	Trace ^C^
		Pulp	2.09 ± 0.05 ^B^	Not detected ^C^	3.24 ± 0.12 ^A^	Not detected ^C^	Not detected ^C^
**21**	Poncirin	Peel	221 ± 12 ^B^	15.6 ± 1.3 ^D^	767 ± 68 ^A^	120 ± 9.0 ^C^	3.68 ± 0.34 ^D^
		Pulp	142 ± 5 ^B^	4.82 ± 0.20 ^D^	371 ± 6.6 ^A^	15.8 ± 0.2 ^C^	0.295 ± 0.006 ^D^
	Total flavonoids and phenolic acids	Peel	3432 ± 239	1519 ± 121	2360 ± 220	2306 ± 117	2635 ± 193
		Pulp	612 ± 26	361 ± 19	896 ± 43	301 ± 4.0	502 ± 20
	*Anthocyanins*						
**Peak**	**Compound**		**Collette**	**Yellow Sunshine**	**Pink Ice**	**Red**	***Faustrime***
**22**	Cyanidin 3-*O*-glucoside	Peel	20.0 ± 0.5 ^A^	nd	nd	12.3 ± 0.2 ^B^	nd
		Pulp	nd	nd	0.925 ± 0.07 ^B^	1.44 ± 0.09 ^A^	nd
**23**	Petunidin rhamnosyldiglucoside	Peel	Trace ^B^	nd	Nd	20.4 ± 0.3 ^A^	nd
		Pulp	nd	nd	Trace ^B^	3.41 ± 0.10 ^A^	nd
**24**	Cyanidin 3-(6′′-malonylglucoside)	Peel	20.3 ± 1.0 ^A^	nd	nd	17.1 ± 0.3 ^B^	nd
		Pulp	nd	nd	Trace ^B^	0.62 ± 0.14 ^A^	nd
**25**	Delfinidin rhamnosylglucoside	Peel	71.1 ± 1.0 ^A^	nd	nd	3.80 ± 0.15 ^B^	nd
		Pulp	nd	nd	1.98 ± 0.01 ^A^	Not detected ^B^	nd
**26**	Peonidin 3-(6″-malonylglucoside)	Peel	31.8 ± 0.7 ^B^	nd	nd	42.6 ± 1.3 ^A^	nd
		Pulp	nd	nd	1.47 ± 0.03 ^B^	2.79 ± 0.23 ^A^	nd
	Total anthocyanins	Peel	143.2 ± 3.2	nd	nd	96.2 ± 2.3	nd
		Pulp	nd	nd	4.38 ± 0.11	8.26 ± 0.56	nd
	Total phenols	Peel	3575 ± 242	1519 ± 121	2360 ± 220	2402 ± 119	2635 ± 193
		Pulp	612 ± 26	361 ± 19	900 ± 43	309 ± 4.6	502 ± 20

^a^ Compound numbers correspond to the peak numbers in Figure 1. The superscript uppercase letters (A–E) indicate statistically significant differences among the varieties.

**Table 4:** Complete composition of the peel and pulp essential oils hydrodistilled from all of the analysed *Citrus australasica* varieties (Collette, Pink Ice, Red, and Yellow Sunshine), and for the *Faustrime* hybrid.

Compounds	l.r.i. ^a^	Relative Abundance (%) ± SD									
		Collette		Pink Ice		Red		Yellow Sunshine		Faustrime	
		Peel	Pulp	Peel	Pulp	Peel	Pulp	Peel	Pulp	Peel	Pulp
α-Thujene	931	-	-	-	-	-	-	-	-	0.1 ± 0.01	-
α-Pinene	941	0.7 ± 0.02	-	0.4 ± 0.05	-	0.1 ± 0.00	0.2 ± 0.24	-	-	1.1 ± 0.08	1.0 ± 0.08
Sabinene	976	1.7 ± 0.04	-	1.7 ± 0.13	-	-	-	-	-	0.3 ± 0.02	0.2 ± 0.02
β-Pinene	982	0.4 ± 0.02	-	0.1 ± 0.01	-	-	-	-	-	0.5 ± 0.03	0.2 ± 0.02
Myrcene	993	1.0 ± 0.03	-	0.7 ± 0.04	-	1.0 ± 0.03	-	0.3 ± 0.08	-	1.0 ± 0.06	1.0 ± 0.03
Octanal	1001	-	-	-	-	-	-	-	-	0.6 ± 0.03	-
α-Phellandrene	1005	4.1 ± 0.57	-	3.1 ± 0.18	-	0.1 ± 0.01	-	-	-	5.2 ± 0.30	7.2 ± 0.41
δ-3-Carene	1011	0.2 ± 0.04	-	0.1 ± 0.01	-	0.5 ± 0.01	-	-	-	0.3 ± 0.02	0.2 ± 0.01
α-Terpinene	1018	1.3 ± 0.16	-	3.7 ± 0.21	-	-	-	-	-	0.8 ± 0.04	2.1 ± 0.09
*p*-Cymene	1027	0.3 ± 0.02	-	0.2 ± 0.01	-	-	-	-	-	1.0 ± 0.05	0.4 ± 0.04
Limonene	1032	42.4 ± 5.64	1.4 ± 0.48	26.5 ± 1.75	1.0 ± 0.54	73.6 ± 4.41	-	40.0 ± 2.47	0.4 ± 0.11	31.5 ± 1.86	48.3 ± 3.71
1,8-Cineole	1034	0.1 ± 0.01	-	-	-	-	-	-	-	-	-
(*Z*)-β-Ocimene	1042	0.3 ± 0.01	-	0.3 ± 0.01	-	1.2 ± 0.01	-	0.5 ± 0.05	-	0.7 ± 0.04	0.3 ± 0.01
(*E*)-β-Ocimene	1052	0.1 ± 0.01	-	0.2 ± 0.01	-	0.5 ± 0.00	-	0.4 ± 0.11	-	0.2 ± 0.01	0.1 ± 0.00
γ-Terpinene	1062	14.2 ± 1.96	-	7.3 ± 0.25	-	0.3 ± 0.01	-	0.4 ± 0.11	-	11.6 ± 0.5	10.8 ± 0.47
Terpinolene	1088	1.6 ± 0.24	-	2.0 ± 0.01	-	0.4 ± 0.01	-	-	-	1.2 ± 0.02	0.9 ± 0.01
Linalool	1101	0.9 ± 0.17	1.1 ± 0.11	0.3 ± 0.00	-	-	-	-	0.2 ± 0.04	2.6 ± 0.04	0.7 ± 0.01
Nonanal	1104	-	-	-	-	-	-	-	-	0.2 ± 0.01	-
*cis*-*p*-Menth-2-en-1-ol	1124	0.6 ± 0.03	1.2 ± 0.11	1.7 ± 0.05	-	-	-	-	-	1.1 ± 0.00	0.3 ± 0.00
*trans*-*p*-Menth-2-en-1-ol	1140	0.4 ± 0.03	1.2 ± 0.45	1.3 ± 0.04	-	-	-	-	-	0.8 ± 0.01	0.2 ± 0.01
β-Terpineol	1153	-	-	-	-	-	-	-	-	1.6 ± 0.02	0.1 ± 0.01
Menthone	1154	5.1 ± 0.14	1.0 ± 0.06	-	-	-	-	-	-	-	0.3 ± 0.01
Citronellal	1155	0.7 ± 0.04	-	-	-	1.7 ± 0.06	-	1.1 ± 0.12	-	9.4 ± 0.05	-
*iso*Borneol	1156	-	-	-	-	-	-	-	-	0.9 ± 0.03	-
*iso*Menthone	1164	-	-	2.6 ± 0.09	-	-	-	-	-	1.4 ± 0.03	-
4-Terpineol	1178	8.4 ± 0.23	12.0 ± 1.07	38.3 ± 0.81	19.3 ± 4.14	-	-	-	-	2.1 ± 0.06	0.4 ± 0.02
*iso*Menthol	1179	-	-	0.2 ± 0.01	-	-	-	-	-	-	-
Cryptone	1187	-	-	-	-	-	-	-	-	0.2 ± 0.01	-
α-Terpineol	1189	1.2 ± 0.06	2.5 ± 0.31	1.9 ± 0.11	-	-	-	-	0.3 ± 0.04	3.1 ± 0.11	0.7 ± 0.06
*cis*-Piperitol	1195	0.2 ± 0.02	-	0.6 ± 0.04	-	-	-	-	-	0.3 ± 0.02	-
Decanal	1204	-	-	0.3 ± 0.04	-	-	-	-	-	0.5 ± 0.02	0.7 ± 0.06
*trans*-Piperitol	1207	0.4 ± 0.05	0.5 ± 0.20	1.0 ± 0.05	-	-	-	-	-	0.6 ± 0.04	0.2 ± 0.01
Citronellol	1230	1.1 ± 0.12	-	0.1 ± 0.08	-	0.4 ± 0.13	-	-	-	1.7 ± 0.11	-
Neral	1240	-	-	-	-	-	-	-	-	1.0 ± 0.07	-
Piperitone	1252	2.5 ± 0.15	2.7 ± 0.25	0.1 ± 0.07	-	-	-	-	-	6.9 ± 0.48	1.7 ± 0.16
(*E*)-2-Decenal	1260	-	-	-	-	-	-	-	-	0.1 ± 0.01	-
Phellandral	1272	-	-	-	-	-	-	-	-	0.1 ± 0.01	-
*trans*-Citral	1273	-	-	-	-	-	-	-	-	1.6 ± 0.13	-
*n*-Tridecane	1300	-	-	-	-	-	-	-	-	-	0.3 ± 0.04
Undecanal	1306	-	-	-	-	-	-	-	-	-	0.1 ± 0.01
δ-Elemene	1340	0.1 ± 0.01	0.4 ± 0.50	-	-	0.3 ± 0.07	1.8 ± 0.84	2.0 ± 0.00	0.6 ± 0.02	-	-
Citronellyl acetate	1354	-	-	-	-	-	-	-	-	0.2 ± 0.04	0.1 ± 0.02
Eugenol	1358	-	-	-	1.1 ± 0.04	-	-	-	0.4 ± 0.14	-	-
β-Elemene	1392	-	-	-	-	-	-	0.4 ± 0.00	-	-	-
1-Tetradecene	1392	-	-	-	-	-	-	-	-	-	0.1 ± 0.08
*n*-Tetradecane	1400	-	-	-	-	-	-	-	-	-	0.1 ± 0.09
Dodecanal	1408	-	-	0.1 ± 0.07	-	-	-	-	-	-	0.4 ± 0.11
*cis*-α-Bergamotene	1416	-	-	-	-	-	-	-	-	-	0.1 ± 0.02
β-Caryophyllene	1420	0.7 ± 0.11	21.3 ± 1.05	0.4 ± 0.09	14.1 ± 1.00	0.2 ± 0.04	6.9 ± 1.20	-	0.6 ± 0.22	0.8 ± 0.16	2.3 ± 0.39
*trans*-α-Bergamotene	1438	-	1.2 ± 0.37	-	2.3 ± 0.01	0.2 ± 0.04	1.9 ± 0.56	-	0.9 ± 0.25	1.5 ± 0.31	2.9 ± 0.48
α-Humulene	1456	-	8.4 ± 0.13	0.6 ± 0.14	12.7 ± 0.52	0.2 ± 0.06	4.0 ± 0.37	-	0.3 ± 0.06	1.0 ± 0.21	4.6 ± 0.69
(*E*)-β-Farnesene	1460	-	-	-	-	-	0.2 ± 0.23	-	-	-	0.2 ± 0.04
β-Santalene	1463	-	-	-	-	-	-	-	-	-	0.1 ± 0.02
γ-Gurjunene	1474	0.5 ± 0.72	0.9 ± 0.25	-	-	-	-	-	-	-	-
γ-Muurolene	1477	-	0.7 ± 0.25	-	-	-	-	-	-	-	-
Germacrene D	1478	0.3 ± 0.06	-	-	-	0.3 ± 0.07	2.7 ± 0.04	2.6 ± 0.05	1.0 ± 0.08	-	0.2 ± 0.01
β-Chamigrene	1485	-	-	-	-	-	-	-	-	-	0.1 ± 0.03
δ-Selinene	1490	-	-	-	-	0.1 ± 0.07	1.0 ± 0.11	-	1.2 ± 0.11	-	-
Bicyclogermacrene	1496	4.9 ± 0.62	10.9 ± 0.33	1.9 ± 0.40	9.8 ± 0.74	6.9 ± 1.10	28 ± 1.61	39.8 ± 2.24	20.3 ± 0.07	0.8 ± 0.20	1.0 ± 0.20
*n*-Pentadecane	1500	-	-	-	-	-	-	-	-	-	1.1 ± 0.25
(Z)-α-Bisabolene	1504	-	-	-	-	-	-	-	-	-	0.4 ± 0.09
α-Bulnesene	1505	-	-	-	-	-	-	0.9 ± 0.07	0.3 ± 0.03	-	-
Germacrene A	1506	-	-	-	-	0.2 ± 0.03	-	-	-	-	-
α-Chamigrene	1508	-	-	-	-	-	2.4 ± 0.36	2.5 ± 0.14	-	-	-
β-Bisabolene	1509	0.8 ± 0.12	4.8 ± 0.4	0.7 ± 0.18	9.2 ± 0.35	2.0 ± 0.42	24.6 ± 0.14	-	-	2.6 ± 0.57	5.6 ± 1.05
*trans*-γ-Cadinene	1513	-	-	-	-	-	-	-	0.3 ± 0.06	-	-
(*Z*)-γ-Bisabolene	1515	-	-	-	-	-	-	-	-	-	0.1 ± 0.03
Selina-3,7(11)-diene	1542	-	-	-	-	-	-	-	-	-	0.1 ± 0.07
Germacrene B	1554	-	-	-	-	0.6 ± 0.15	5.2 ± 0.01	0.9 ± 0.02	0.2 ± 0.25	-	0.5 ± 0.11
Palustrol	1568	-	1.3 ± 0.06	0.2 ± 0.04	-	0.6 ± 0.12	1.7 ± 0.79	-	6.0 ± 0.00	-	-
Spathulenol	1576	-	-	-	-	0.1 ± 0.04	-	1.1 ± 0.15	-	-	-
Caryophyllene oxide	1581	-	-	-	15.6 ± 1.29	-	-	-	-	-	-
Globulol	1583	0.6 ± 0.12	9.5 ± 0.13	0.5 ± 0.13	-	1.8 ± 0.37	3.8 ± 0.99	2.9 ± 0.23	14.5± 0.15	-	0.1 ± 0.08
Viridiflorol	1590	0.7 ± 0.12	7.9 ± 0.30	0.4 ± 0.13	5.8 ± 1.58	1.7 ± 0.34	10.9 ± 0.21	1.2 ± 0.21	19.9 ± 0.01	-	-
Guaiol	1595	0.2 ± 0.03	1.1 ± 0.35	0.2 ± 0.03	-	0.5 ± 0.11	3.2 ± 0.05	0.3 ± 0.01	11.9 ± 0.71	-	-
Cedrol	1596	0.3 ± 0.12	1.4 ± 0.42	0.3 ± 0.08	-	0.8 ± 0.17	1.0 ± 1.45	0.8 ± 0.07	8.5 ± 0.08	-	-
*n*-Hexadecane	1600	-	-	-	-	-	-	-	-	-	0.6 ± 0.22
5-*epi*-7-*epi*-α-Eudesmol	1603	-	-	-	-	-	-	-	2.2 ± 0.06	-	-
Humulene epoxide II	1608	-	2.0 ± 0.20	-	4.1 ± 0.83	-	-	-	-	-	-
Selin-6-en-4-ol	1618	-	1.2 ± 0.25	-	-	0.2 ± 0.06	0.5 ± 0.66	-	2.2 ± 0.08	-	-
Caryophylla-4(14),8(15)-dien-5-ol	1637	-	-	-	1.9 ± 0.36	-	-	-	-	-	-
*iso*Spathulenol	1639	-	-	-	-	0.1 ± 0.11	-	-	-	-	-
*epi*-α-Cadinol	1641	-	-	-	3.2 ± 1.03	0.2 ± 0.10	-	0.4 ± 0.05	2.6 ± 0.49	-	-
Cubenol	1643	-	-	-	-	0.2 ± 0.07	-	0.5 ± 0.04	-	-	-
α-Cadinol	1654	-	0.8 ± 0.35	-	-	0.3 ± 0.08	-	0.4 ± 0.10	1.9 ± 0.09	-	-
β-Bisabolol	1672	-	-	-	-	-	-	-	-	0.2 ± 0.25	-
Tetradecanol	1676	-	-	-	-	-	-	-	-	-	0.2 ± 0.08
α-Bisabolol	1683	-	1.4 ± 0.08	-	-	0.2 ± 0.04	-	-	-	0.5 ± 0.18	0.4 ± 0.18
*n*-Heptadecane	1700	-	-	-	-	-	-	-	-	-	0.2 ± 0.08
Monoterpene hydrocarbons		68.2 ± 4.11	1.4 ± 0.48	46.1 ± 2.68	1.0 ± 0.54	77.7 ± 4.37	0.2 ± 0.24	41.6 ± 2.83	0.4 ± 0.11	55.5 ± 3.05	72.8 ± 4.91
Oxygenated monoterpenes		21.7 ± 1.05	22.2 ± 1.05	48.0 ± 1.34	19.3 ± 4.14	2.1 ± 0.18	-	1.1 ± 0.12	0.5 ± 0.08	35.5 ± 1.18	4.7 ± 0.27
Sesquiterpene hydrocarbons		7.3 ± 1.65	48.5 ± 0.41	3.6 ± 0.81	48.1 ± 0.45	10.9 ± 2.04	78.8 ± 3.97	49.2 ± 2.38	25.7 ± 0.03	6.7 ± 1.45	18.2 ± 3.22
Oxygenated sesquiterpenes		1.8 ± 0.39	26.6 ± 0.10	1.6 ± 0.41	30.5 ± 5.09	6.5 ± 1.60	21.1 ± 3.74	7.6 ± 0.60	69.8 ± 1.05	0.6 ± 0.43	0.5 ± 0.25
Phenylpropanoids		-	-	-	1.1 ± 0.04	-	-	-	0.4 ± 0.14	-	-
Non-terpene derivatives		-	-	0.3 ± 0.11	-	-	-	-	-	1.7 ± 0.00	3.7 ± 1.03
Total identified (%)		99.0 ± 1.02	98.6 ± 1.02	99.6 ± 0.01	100 ± 0.01	97.2 ± 0.54	100 ± 0.01	99.5 ± 0.04	96.8 ± 0.74	100 ± 0.01	99.9 ± 0.13

^a^ Linear retention index calculated on a HP-5MS capillary column; - ^:^ Not detected.

## Data Availability

All of the data are contained within the article.

## Supplementary Materials

The following supporting information can be downloaded at: https://www.mdpi.com/article/10.3390/antiox11102047/s1, Table S1: Results of peel and pulp extraction from *Citrus australasica* varieties. Table S2: Concentrations of peel and pulp extracts from *Citrus australasica* varieties applied in the in vitro assay for protection from H_2_O_2_ oxidative stress on Balb/3T3.
