# Supplementary material for: Comparing Metabolomic and Essential Oil Fingerprints of Citrus australasica F. Muell (Finger Lime) Varieties and Their In Vitro Antioxidant Activity
Source: Antioxidants (Basel). 2022 Oct 18;11(10):2047. doi: 10.3390/antiox11102047 (PMC9598366; doi:10.3390/antiox11102047)
Supplement: Supplementary file 1 [file antioxidants-11-02047-s001.zip › antioxidants-1960101-supplementary.pdf]

Comparing metabolomic and essential oil fingerprint of *Citrus australasica* F.Muell (finger lime) varieties and their *in vitro* antioxidant activity

Emily Cioni[1,†], Chiara Migone[1,†], Roberta Ascrizzi[1,2,3], Beatrice Muscatello[1,3], Marinella De Leo[1,2,3*], Anna Maria Piras[1,3], Ylenia Zambito[1,2,3], Guido Flamini[1,2,3], Luisa Pistelli[1,2,3]

[1] *Dipartimento di Farmacia, Università di Pisa, via Bonanno 33, 56126 Pisa, Italy*

[2] *Centro Interdipartimentale di Ricerca "Nutraceutica e Alimentazione per la Salute", via del Borghetto 80, Università di Pisa, 56124 Pisa, Italy*

[3] *Centro per l'Integrazione della Strumentazione dell'Università di Pisa (CISUP), Lungarno Pacinotti 43, 56126 Pisa, Italy*

[†] These authors equally contributed

* Corresponding author.

Full postal address: Dipartimento di Farmacia, Università di Pisa, via Bonanno 33, 56126 Pisa, Italy

E-mail address: marinella.deleo@unipi.it

Phone: 0039 050 2219706

Table S1. Results of peel and pulp extraction from *Citrus australasica* varieties.

	Red	Pink Ice	Collette	Yellow-Sunshine	Faustrime
**Finger lime peel**					
FW (g)	2.9	4.6	5.6	2.4	11.8
DW (g)	0.7	0.9	1.6	0.6	2.5
R_E (mg)	3.2	5.8	21.5	3.0	12.6
R_M (mg)	199.7	297.1	515.1	144.8	663.5
R_M yield (%)	28.5	33.0	32.2	24.1	26.5
**Finger lime pulp**					
FW (g)	7.1	7.0	2.0	3.1	13.2
DW (g)	0.7	0.7	0.3	0.3	1.2
R_E (mg)	1.7	2.2	5.9	1.1	4.5
R_M (mg)	432.4	382.3	121.9	104.3	718.7
R_M yield (%)	61.8	54.6	40.6	34.8	59.9

DW = dry weight; FW = fresh weight; R_E = *n*-hexane extract; R_M = methanolic extract.

Table S2. Concentrations of peel and pulp extracts from *Citrus australasica* varieties applied in the *in vitro* assay of protection from H_2O_2 oxidative stress on Balb/3T3.

	Finger lime peel				
	**Red**	**Pink Ice**	**Collette**	**Yellow Sunshine**	**Faustrime**
	31.5	40.3	43.6	35.4	54.2
Concentration	15.8	20.2	21.8	17.7	27.1
(µg/mL)	7.9	10.1	10.9	8.9	13.6
	**Finger lime pulp**				
	**Red**	**Pink Ice**	**Collette**	**Yellow Sunshine**	**Faustrime**
	124.6	85.8	73.1	132.8	191.9
Concentration	62.3	42.9	36.6	66.4	96.0
(µg/mL)	31.2	21.5	18.3	33.2	48.0
